# Dough Rheology, Physicochemical and Sensory Qualities of Muffins Made From Wheat–Partially Defatted Peanut Composite Flour

**DOI:** 10.1155/ijfo/6635106

**Published:** 2025-06-25

**Authors:** Tengetile Mkoko, Solomon W. Kidane, Jeremiah S. Shelembe, Thabile P. Nkambule, Muzi M. Dlamini, Tebesutfu B. Nkhambule

**Affiliations:** Department of Food and Nutrition Sciences, Faculty of Consumer Sciences, University of Eswatini, Luyengo, Eswatini

**Keywords:** bread, peanut, rheology, sensory evaluation

## Abstract

Peanuts are nutritious, containing antioxidants and health benefiting and promoting bioactives. Moreover, they have excellent amino acid profiles composed of 20 amino acids making them an important ingredient to enhance the quality baked products like muffins. The influence of blend proportion of partially defatted peanut flour (PDPF) at the level of 10%, 15%, 20%, and 25% on dough rheology and quality attributes of muffins made from wheat–PDPF composite flour was studied. The Farinograph and Alveograph properties were significantly (*p* < 0.05) affected due to the proportion of PDPF. The dough development time (DDT) increased, whereas the water absorption capacity (WAC), stability (*S*), tenacity (*p*), dough strength (*W*), extensibility (*L*), swelling index (*G*), elasticity index (Ie), and configuration ratio (*P*/*L*) significantly (*p* < 0.05) decreased with increase in the relative percentage of PDPF. The protein, fat, fiber, and ash content of muffins also increased with increase in the proportion of PDPF. The crumb and crust colors were also significantly influenced where the crust and crumb lightness (*L*∗) value decreased from 60.3 to 53.8 and from 62.5 to 51.2 with increase in PDPF, respectively. Incorporation of the PDPF improved the sensory evaluation scores for texture, taste, color, flavor, and overall acceptability. In view of relatively high dough rheological properties including WAC (59.9%), stability (10.8 min), *L* (56 mm), *W* (108 × 10^−4^ J), and high contents of protein (10.5%), fiber (1.8%), and ash (1.9%) coupled with high sensory taste (7.2) and overall acceptability (7.3) scores, PDPF can be incorporated up to 15% for acceptable muffins.

## 1. Introduction

Interest in fortifying food products with the aim at enhancing nutrition and health benefits is growing recently [1, 2]. This is in response to the demand by consumers for nutritious and health-benefitting products. Peanuts are nutritious and also contain antioxidant components, health benefiting, and promoting bioactives [3–6]. The nutritional composition of peanuts has been reported as protein (20%–42.1%), lipids (41.5%–50%), fiber 3.7%–8.5%, and ash (2.5%–3.8) [3, 6–10]. Moreover, peanuts have excellent amino acid profiles composed of 20 amino acids. The absence of cholesterol, coupled with the presence of monounsaturated fatty acid (MUFA) and polyunsaturated fatty acid (PUFA) in significant amounts, makes the lipid desirable and healthy [5]. The nutritional quality of baked products can be improved significantly by the incorporation of nuts since they contain an appreciable quantity of protein, fiber, unsaturated fats, and vitamins, particularly E, A, B_1_, and B_2_ [3, 10]. Earlier reports indicated that peanuts and peanut-based products exhibit health benefits [3, 4, 11]. Consumption of peanuts has been associated with a low risk of developing Type II diabetes and also the prevention of cardiovascular diseases [3, 12]. Peanuts are also reported to have antitumor, antioxidant, and antifungal properties [4]. The hypogin, a novel antifungal peptide from peanuts, exhibited antifungal activity. The inhibitory effect on the growth of *Coprinus comatus* and *Mycosphaerella arachidicola* was especially conspicuous [13].

Various peanut-based products, ranging from peanut butter to blends with other ingredients, are available worldwide [3]. Baked products like protein bars, confections, cakes, cookies, snacks, crackers, biscuits, flat bread, and other products including flour dispersions and pasta have been produced using peanut flours [5, 7, 11, 14–18]. Previous studies have demonstrated the potential of partially replacing wheat flour with peanut-based ingredients in baked products to enhance their nutritional and sensory qualities. For example, wheat flour replaced by 15% and 20% reduced-fat peanut flour and defatted peanut meal flour, respectively, resulted in acceptable bread [14, 18]. The results indicated that peanut flour can be used successfully to improve the nutritional and sensory qualities of bread. Earlier studies have also indicated that it is possible to replace wheat flour with peanut and other nuts paste. The results of these studies indicated that breads added with 10% and 15% nut paste showed the highest intent for consumption, persistence, and texture values [19]. In a related study, loaf volume increased when wheat flour was replaced by 5%–10% of nut paste [8]. Improved nutritional quality in terms of the protein quality, in vitro protein digestibility, dietary fiber content, and lowered glycaemic index has been observed in flat bread made by replacing wheat flour with defatted peanut flour up to 30% [5]. The effect of peanut butter and peanut flour on the physical properties of muffins has been studied, and the results indicated up to 32% by weight of peanut flour can be incorporated [20]. Replacement of wheat flour by peanut flour significantly (*p* < 0.05) increased tenderness of muffins. As peanut flour proportion was increased, the outer surface of muffins became browner, whereas increased peanut butter gave rise to a more intense hue. Internal crumb color became darker and more intense brown as both peanut flour and peanut butter were increased. However, the study did not involve sensory evaluation and rheological properties of the dough [20].

Though there are varieties of peanut-based products, there is still a need to diversify the utilization of this important legume. Specifically, information on the possibilities of using partially defatted peanut flour (PDPF) for the production of muffins has not been reported. Information on the possibility of incorporating peanut flour to make muffins is scant in the literature. Moreover, the influence of blending peanut flour with wheat flour on dough rheology, physicochemical properties, and sensory qualities of muffins is nonexistent in the literature. This study was aimed at determining the influence of the proportion of PDPF on dough rheological properties and physicochemical properties and the acceptability of wheat–PDPF composite flour muffins.

## 2. Materials and Methods

### 2.1. Materials

White bread wheat flour was supplied by Premier Mill Company, Matsapha, Eswatini, with composition of 11.8% protein, 0.9% total fat, and 4.1% fiber. Hexane was sourced from MCB Laboratory Solutions, Matsapha, Eswatini. The peanuts were sourced from local market. Whole milk produced by a dairy processing plant (Lactalis Eswatini (PTY) Ltd.) was used. Canola cooking oil was sourced from the local market.

### 2.2. PDPF Preparation

The raw peanuts were cleaned to remove foreign materials and defective nuts. Roasting was done at 190°C for 20 min (Incotherm, Labotech 926, Telaangana, India) to ease the removal of the skins by modifying the method used by Chukwumah et al. [21]. Roasted peanuts were ground into flour using a blender (KM-1500 MRC, Laboratories, Holon, Israel). Defatting was done by soaking the roasted peanut flour in hexane for 36 h followed by filtering and spreading in fume hood to evaporate the remaining hexane for 24 h. The PDPF was used immediately.

### 2.3. Composite Flour Preparation

A total of five formulations including the control (100% wheat flour, 100 W) and composite flour with 10% PDPF (90W10PDPF), 15% PDPF (85W15PDPF), 20%PDPF (80W20PDPF), and 25% PDPF (75W25PDPF) were prepared. These proportions were selected based on ranges used in previous similar studies [14, 16].

### 2.4. Rheological Properties of Dough

Water absorption capacity (WAC), dough development time (DDT), and stability were measured using Farinograph (Farinograph AT, Villeneuve, France) based on AACC Method 54-21 constant dough method [22]. The Alveograph parameters were measured in terms of pressure (tenacity, *P*), swelling index (*G*), extensibility (*L*), elasticity index (Ie), deformation energy or dough strength (*W*), and extensibility ratio (configuration ratio, *P*/*L*) using Alveograph (Chopin Technologies, Villeneuve, France) based on AACC Method 54-30 [22]. The moisture content of flour was determined prior to the Alveograph test in order to determine the amount of NaCl to be added using AACC Method 44-15.02 [22].

### 2.5. Muffin Baking

The formulations were made of 400 g composite flour, 150 g white sugar, 2 eggs, 250 mL whole milk, 130 g canola cooking oil, and 20 g baking powder. Eggs were beaten in a bowl together with milk and sugar. The dry ingredients were then added bit by bit while folding in manually until the right consistency was achieved. Baking was done at 180°C for 20 min (Macadams Deck Oven, Macadams, Western Cape, South Africa) followed by cooling the muffins at ambient temperature before quality evaluation were conducted [23]. Quality evaluation was done immediately after the muffins were cooled down.

### 2.6. Analysis Proximate Composition

The proximate composition of the PDPF and muffins made from different formulations was analyzed for moisture, protein, ash, fat, and fiber content. Moisture and ash were measured using AACC [22] with Methods 934.01 and 942.05, respectively. Total dietary fiber and proteins were determined according to AOAC [24] Methods 985.29 and 968.06, respectively. The Soxhlet method using hexane was employed to determine the fat content [25].

### 2.7. Physical Properties of Muffin

The muffin physical properties were assessed in terms of specific volume, height, weight, volume, baking loss, and crust and crumb color. Analytical scale (E.I.M series N17250, Milton Keynes, United Kingdom) was used to determine weight. Rapeseed displacement method [22] (Method 10-05.1) was used to measure volume, and the specific volume was determined as the ratio of volume to weight of muffins. The height of the muffin was determined by using a Vernier caliper. Color was measured in terms of *L*∗ for lightness, *a*∗, and *b*∗ for chromaticity coordinates (+*a*∗ = red to −*a*∗ = green; −*b*∗ = blue to +*b*∗ = yellow) using colorimeter (CLRM-310, MRC, Israel). The percentage of the difference between the weights of fresh dough and muffin weight after baking was used as a measure of baking loss [18].

### 2.8. Sensory Evaluation

A sensory evaluation was conducted to evaluate flavor, taste, texture, color, and overall acceptance (OAC) of muffin sample. A total of 50 panelists aged between 20 and 30 evaluated samples on a 9-point hedonic scale ranging from 9 = *like extremely* to 1 = *dislike extremely*. The assessors were given proper orientation before the sensory sessions commenced. The samples were presented in randomized order. The sensory evaluation is done in individual booths where there is consistent light and good ventilation.

### 2.9. Data Analysis

Comparison of mean was conducted using one-way ANOVA by IBM SPSS Version 20 (IBM SPSS Inc, Illinois, United States). Each observation was replicated three times and mean separation was done using Duncan's multiple range test at (*p* < 0.05). Outlier box plot method was employed to identify outliers from the sensory data. Principal component analysis was done using SAS-JMP Pro 16 (SAS Institute, Inc, North Carolina, United States) to assess associations between variables and variability among samples.

## 3. Results and Discussion

### 3.1. Proximate Composition of PDPF

The proximate composition of the PDPF was found to be 19.33% moisture content, 31.48% fat, 28.36% protein, 6.74% fiber, and 3.02% ash. The values obtained in this study were comparable for PDPFs in other studies. The fat content obtained in this study is higher than the 27.5% fat content of PDPF prepared using mechanical press [14]. The difference in fat content could be attributed to the method of defatting. The fat content obtained in the PDPF was lower than the fat content from whole peanut flours, which is in the ranges of 45%–50% [26, 27]. The fiber and ash contents obtained in the present study are within the range as those observed in similar studies and confirm that peanut supplies fiber and minerals [3, 27]. Total dietary fiber ranging from 9.6% to 13% and ash content from 2.0% to 2.3% were previously reported for different peanut varieties [26].

### 3.2. Dough Rheology

#### 3.2.1. Farinograph Parameters

#### 3.2.2. WAC

The WAC was significantly (*p* < 0.05) decreased from 59.65% to 55.15% with increase in PDPF composition from 0% to 25% (Table 1). Nonetheless, the samples that contain PDPF were not significantly different (*p* < 0.05). A significant (*p* < 0.05) decrease in WAC from 54.35% to 45.69% with increase in nut paste composition from 0% to 15% has been reported in an earlier study. The observed trend was attributed to the presence of fat that reduced water absorption and increased the DDT [8]. Fat and oils weaken the dough strength, thus delaying in the development of gluten, resulting long development time [28], and fats also affect dough permeability to water [29]. In similar studies, increased replacement of wheat flour by other legumes like germinated chickpea flour, lupin flour, and pea flour resulted in decreased WAC [30, 31], whereas replacement of wheat flour by full fat soy flour from 0% to 12% did not bring a significant influence in WAC [32]. The reduction in WAC of samples compared to the control could be attributed to roasting of peanuts which denatures the protein and expose more hydrophobic sites [33]. In contrast, however, other studies reported increased WAC as a result of increased native protein and fiber content [16, 34].

##### 3.2.2.1. DDT

The DDT significantly (*p* < 0.05) increased from 2.1 for the control to 5.6 min with increase in PDPF up to 25% (Table 1). The DDT is an indicator of flour strength where higher values suggesting stronger dough [35]. An earlier study indicated that increase in nut paste quantity from 0% to 15% resulted increased DDT from 124 to 211 s in wheat–nut paste blend dough [8]. An increase in peanut flour percentage from 0% to 20% brought about increase in DDT from 2.5 to 6 min in white flour dough and from 6 to 7 min in brown bread flour dough [16]. Lentil flour addition from 0% to 24% also resulted in increase in DDT from 1.45 to 5.15 min [36]. Increase in the DDT is associated with the increase fiber content [8, 16, 34, 37], and peanut is known as good source of fiber. Several studies indicated that addition of protein-rich flours prolongs DDT. Increase in DDT from 1.3 to 6.8 min and from 1.8 to 4.5 min has been reported with increase in soya bean and mushroom flour proportion from 5% to 20%, respectively, in wheat-based composite flours [38]. DDT has also been reported to be prolonged due to addition of soy flour and soy protein isolate in wheat–soy composite flour [32, 39].

##### 3.2.2.2. Stability

The PDPF proportion significantly (*p* < 0.05) affected the stability where an increase in PDPF proportion from 0% to 25% resulted in a decrease in the dough stability from 11.4 to 4.04 min (Table 1). The stability value gives some indication of the mixing tolerance and flour strength [40]. Wheat dough with high protein content exhibits high stability compared to low and medium protein content. Good bread making flour is characterized by high water absorption, long mixing time, and good tolerance to overmixing [41]. An increase in PDPF proportion lowers the bread making quality, which could be attributed to the dilution of gluten protein. Similar trends were observed with the incorporation of green pea, yellow pea, red lentil, and commercial chickpea [40]. The decrease in the stability value with an increase in the proportion of PDPF could also be attributed to the increased fiber content from the PDPF. Fiber particles induce notable disruption of the gluten network continuity and thus weaken its stability, resulting in reduced stability time [42]. A decrease in stability with an increase in fiber proportion has been observed in an earlier study [34].

#### 3.2.3. Alveograph Parameters

##### 3.2.3.1. Pressure (Tenacity) (mmH_2_O)

The tenacity value significantly (*p* < 0.05) decreased from 107.5 mmH_2_O for the control to 45 mmH_2_O for sample with 25% PDPF (Table 1). Tenacity is the maximum over pressure in mmH_2_O needed to inflate the dough bubble and is associated with dough resistance to deformation and also tensile strength [43]. Addition of nongluten protein weakens dough due to competition for water between the nongluten and gluten protein [32]. Moreover, the reduction in tenacity could be attributed to the fat contributed by PDPF that has been reported to have a lubricating and softening effect. Fat coats the surface of the flour particles inhibiting the development of the gluten proteins. The free fat therefore disrupts the gluten network resulting in softer doughs [44]. Tenacity has been related to WAC where increase in WAC is associated with increase in tenacity [43]. Increase in the proportion of native peanut flour proportion from 10% to 20% increased *P* from 212.6 to 244.6 mmH_2_O [16]. Increase in nut paste proportion from 0% to 15% in wheat–nut paste composite dough brought about increased tenacity from 68 to 99.46 mmH_2_O which was attributed to increased fiber content [8, 37]. Fibers exhibit greater water absorption than gluten, and water migration can occur during mixing. This involves partial dehydration of the flour components while the fiber components are hydrated. The reduced availability of water for the gluten network causes conformational changes in the gluten proteins, making the dough stiffer and more resistant to mixing, thereby increasing consistency. The competition for water and the resulting stiffening of the dough causes increased tenacity [45, 46].

##### 3.2.3.2. Extensibility (*L*)

The dough extensibility significantly (*p* < 0.05) decreased from 60 mm for the control to 38 mm for samples with 25% PDPF (Table 1). It is also referred to as dough biaxial extensibility and is an indicator of the extent to which a dough piece can extend without breaking. *L* is associated with the quality parameters like volume and crumb structure [43]. Earlier studies also indicated that the replacement of wheat protein with sources like lupin and pumpkin seed flour brought about a decrease in extensibility. Extensibility was reduced from 62.8 to 23.2 mm and from 62.8 to 25.6 mm due to the replacement of wheat with 25% ungerminated and germinated pumpkin seeds [47]. Similarly, the replacement of wheat by germinated lupin flour up to 25% resulted in a reduction of extensibility from 62.8 to about 14 mm [30]. These changes are associated with the weakening of the gluten matrix as a result of wheat flour replacement [48]. Reduction in extensibility was observed when defatted soy flour was blended with wheat flour. However, increased replacement of wheat with undefatted peanut and undefatted soy flour increased extensibility [8, 16, 32]. An increase in the proportion of defatted soy flour resulted in sticky dough difficult to handle [32]. An increase in the proportion of other undefatted nut paste resulted in increased extensibility [8]. The replacement of wheat flour by soy protein isolates also reduced dough extensibility [32].

##### 3.2.3.3. Swelling Index (*G*)

The swelling index decreased significantly (*p* < 0.05) from 17.2 for the control to 13.75 for the dough with 25% PDPF (Table 1). The swelling index is related to the extensibility of doughs [43, 49]. The decrease in *G* with increase in the proportion of PDPF could be due to replacement of gluten with nongluten protein and also increase in the fiber content with a higher proportion of PDPF. Dough from whole wheat flour has a low G value compared to refined wheat flour, which could be associated with the high fiber content in whole wheat flour [49].

##### 3.2.3.4. Deformation Energy (*W*)

A significant (*p* < 0.05) reduction in *W* from 241.5 × 10^−4^ J to 62 × 10^−4^ J was observed due to changes in the proportion of PDPF from 0% to 25% (Table 1). *W* is expressed as the energy required to inflate the dough bubble until rupture and is also associated with flour strength, dough strength, baking strength, or flour protein strength [43]. The decrease in *W* due to increase in PDPF is associated to the relative decrease in gluten since *W* is a function of the quantity and quality of gluten [43, 50]. A decrease in *W* from 310 × 10^−4^ J to 8.9 × 10^−4^ J and from 310 × 10^−4^ J to 151 × 10^−4^ J has been observed for dough made by replacing wheat with 0%–25% of germinated and ungerminated pumpkin seed powder, respectively [47]. Bread fours are categorized by *W* values. Dough with *W* < 100 × 10^−4^ J is good for cookie baking, and those with *W* > 100 × 10^−4^ J can be used for several types of bread making [51]. A significant and positive association has been reported between *W* and bread volume [52]. The results in this study suggest that PDPF can be incorporated up to 15%.

##### 3.2.3.5. Elasticity Index

The PDPF significantly (*p* < 0.05) affected the value of the elasticity index (Ie). The Ie was 54.15% for the control (100% wheat) and 24.6% for the dough with 25% PDPF (Table 1). Ie is used as an indication of the dough's elasticity. Flour constituents (proportion of protein, fiber, and fat) and the addition of other ingredients or flours to the wheat flour affect the value of Ie [43]. Thus, the decrease in Ie with increase in PDPF could be attributed to the replacement of wheat protein with a nongluten protein [47, 53]. The increase in the replacement of wheat flour by pea flour from 2.5 % to 20% resulted in a decrease Ie from 145 to 90 mm, which has been observed [31]. A significant (*p* < 0.0001) positive association (*r* = 0.80) has been reported between bread volume and Ie from 19 European commercial wheat flours [53]. Very high and very low values are undesirable for bread making, where high Ie values result in hard dough that is difficult to elongate and shrink. Values in the range of 43%–60% were reported for 19 European commercial wheat flours [53] suggesting the possibility of incorporating PDPF up to 15%. A positive association has been reported between Ie and the height-to-width ratio of rolls made from 37 commercial wheat flours from 14 mills located in seven European countries [54].

##### 3.2.3.6. Configuration Ratio

The configuration ratio (*P*/*L*) value significantly (*p* < 0.05) decreased with an increase in the PDPF (Table 1). The maximum *P*/*L* was 1.80 for the control, whereas the minimum was 1.04 for the 10% PDPF. The *P*/*L* is the ratio between the maximum overpressure and the length of the curve. This ratio is associated with dough resistance and extensibility properties, where high *P*/*L* is linked with a resistant and inextensible dough, and low *P*/*L* is associated with a weak and extensible dough [43]. The decrease in *P*/*L* up to 15% PDPF indicates the decrease in *P* is more pronounced than the decrease in *L*. The *P*/*L* ratio, however, increased though not significantly (*p* < 0.05), with a further increase in PDPF indicating a fall in extensibility. The change in *P*/*L* ratio could be the result of the interaction between fiber and wheat protein and weakening of wheat protein due to peanut protein [37]. *P*/*L* is usually used in conjunction with *W* to evaluate the quality of flour [43]. In view of the results observed, incorporation of PDF up to 15% could result in bread of good quality.

### 3.3. Nutritional Composition of Muffins

The moisture content of muffins was significantly (*p* < 0.05) influenced by the amount of PDFP. The moisture content increased from 24.38% to 26.91% with an increase in PDPF from 10% to 25%. However, the control was not significantly (*p* < 0.05) different from the 25% PDPF samples. The high moisture content might be attributed to the fiber and high nongluten protein content of samples with an increased proportion of PDPF. Fibers and protein are characterized by increased water absorption. Increased fiber and protein content have been associated with high moisture content of baked products [8, 32, 55, 56].

The protein content significantly increased from 9.25% to 13.49%, whereas the fat content increased from 13.3% to 19.5% with increase in the proportion of PDPF. Peanut is a good source of fat and protein; thus, the trend observed in this study was as expected ([3, 14]; A. [9]). The fiber content significantly (*p* < 0.05) increased by 0.336%–2.11% with increasing proportions of PDPF, and no significant (*p* > 0.05) difference was observed between 20% and 25% PDPF (Table 2). The increase in the fiber content due to high proportion of DPDF is linked with the fiber content in PDPF which was found to be 6.74%. Peanut has been reported as good source of fiber [3, 27]. Increase in the crude fiber content from 3.84% db to 5.31% db was also reported with increase reduced-fat peanut flower proportion from 10% to 50% in what–peanut flower composite bread [14]. Similar trend has been observed in cookies made from wheat–peanut cake composite flour cookies [17]. Increase in PDPF content resulted in a significant (*p* < 0.05) increase in ash content (Table 2). There was, however, no significant difference between samples containing 15%, 20% and 25% PDPF. Similar trend has been observed in cookies and biscuits made from wheat–defatted peanut cake flour [3, 17, 57] and bread made from wheat and reduced fat peanut flour [14] and deoiled peanut meal flour [18] indicating that peanut is a good source of minerals. The muffins made from the composite flour resulted increased fiber (from 0.16% to 2.04%), protein (from 9.25% to 13.49%), and ash (1.44% to 2.04%) content. The increment in these components enhances the nutritional and health benefits of the muffins made from the composite flour indicating the potential use of PDPF in the baking industry for manufacturing muffins with better nutrition and health benefit [17].

### 3.4. Physical Properties of Muffins

#### 3.4.1. Height, Weight, Volume, and Specific Volume

The effect of the proportion of PDPF on the height of the muffins is presented in Table 3. The height ranged from 5.33 to 5.71 cm, showing a general increase in muffin height with an increase in the proportion of PDPF, but without an observed significant difference. The amount of PDPF exhibited a significant (*p* < 0.05) influence on volume, where the control had the least volume of 100 cm^3^ but was not significantly different from the other treatments, except the sample with 15% PDPF that had a volume of 145.33 cm^3^. The differences could be attributed to the amount of fat present. There was no significant (*p* < 0.05) difference in the weight as influenced by the proportion of PDPF.

The volume increased with the inclusion of PDPF up to 15%. Further increase in PDPF resulted in a decrease in volume. The specific volume also exhibited a similar trend, where it increased up to 15% inclusion of PDPF then a decrease with further inclusion of 20% and 25% of PDPF (Table 3). The trends observed in this study for volume and specific volume is in agreement with the observation in a similar studies. Addition of peanut paste and other nut pastes increased volume and height of wheat–nut paste composite bread up to 15% where further increase in the proportion of nut paste resulted in a decrease in volume and height [19]. The trend observed could be associated with increased fat content due to addition of nut. Fat improves volume and plays a plasticizer role and imparts softness when used in bread formula [58]. The decrease in volume and specific volume with further addition in nut paste or flour might be due to the weakening and destabilization of the gluten network caused by high quantities of fat [19]. The trends observed in this study are not in line with reports of previous studies for peanut–wheat composite flour bread. A reduction in bread specific volume and volume and has been reported with increase in the proportion of deoiled peanut meal flour, reduced fat peanut flour, and undefatted peanut flour [14, 16, 18].

The amount of PDPF significantly (*p* < 0.05) influenced the baking loss (Table 3). Baking loss in general exhibited a reduction with an increase in the amount of PDPF except for the sample with 25% PDPF. The maximum decrease was for the muffins with 20% PDPF, whereas the maximum loss was from the control. A similar trend was observed where a reduction in baking loss from 36.8% to 31% was noted due to a corresponding inclusion of up to 20% peanut meal flour [18]. The decrease in baking loss was attributed to the amount of protein and fat in the PDPF flour, which could possibly form a complex with water, hence preventing moisture removal during baking. A similar tendency was noted in bread made by replacing wheat flour with chickpea flour [59] and haricot bean and orange flesh sweet potato peel [60]. Baking loss is a function of the water binding capacity of flour, which in turn depends on the protein content, the protein structure, the protein composition, and the starch content [61].

#### 3.4.2. Color Analysis

The lightness (*L*^∗^) value for crust and crumb was affected significantly (*p* < 0.05) by the proportion of PDPF. The *L*^∗^ value in general decreased with the proportion DPDF (Table 4). The highest crust_*L*^∗^ value was 60.3 for the control, and the lowest value was 51.01% for 20% PDPF. Similar trends were noted where increase in the amount of reduced-fat peanut flour resulted in darker loaf [14, 20] which was associated to brown chocolaty color of the peanut flour. Decrease in the lightness with increase the proportion of PDPF could be attributed to the brown color developed during roasting. During roasting, melanoidin pigments are formed due to the reaction between reducing sugars and amino acids during as a result of the Maillard reaction [62]. Similar trends for crust and crumb *L*^∗^ values have been observed for biscuits and bread made with wheat–deoiled peanut meal composite flour [18, 57]. The crust_*a*^∗^ value was also significantly (*p* < 0.05) affected by the proportion of PDPF where increase in PDPF increased the crust_*a*^∗^ values indicating the products exhibited intensified redness. The control exhibited significantly (*p* < 0.05) smaller crust_*a*^∗^ value compared the rest of the treatment. Despite the increase in crust_*a*^∗^ values with increase PDPF proportion, there was no significant (*p* < 0.05) difference in crumb_*a*^∗^ values. Same trend was reported for bread and biscuits made with wheat–deoiled peanut meal composite flour [18, 57].

### 3.5. Sensory Evaluation

The proportion of PDPF significantly (*p* < 0.05) affected the sensory qualities (Table 5). The control exhibited a significantly lower score in all the attributes compared the samples containing various level of PDPF. There was no significant difference (*p* > 0.05) among the samples containing PDPF. The highest color score was observed for samples with 25% PDPF. The crust and crumb colors for samples with PDPF were brownish, whereas the control samples appeared to be lighter as it was evident from the *L*^∗^ values (Table 4). Moreover, the results indicated the taste, favor, texture, and OAC of bread up to 25% were found acceptable by the panelists. The result suggested that brownish color appeared to be attractive to the panelists. In a similar study, bread enriched with peanut and other nut pastes exhibited a nonsignificant (*p* < 0.05) difference in many sensory attributes including flavor, texture, odor, persistence, consumer intent, and overall liking [19]. Increased color score with increase in the proportion of peanut flour was observed in wheat–peanut composite flour bread [14]. No significant (*p* > 0.05) difference in texture, flavor, color, and overall acceptability was observed compared with 100% wheat flour biscuits when wheat was replaced by deoiled peanut meal up to 20% [57]. Similarly inclusion up to 14% defatted peanut cake flour resulted in acceptable cookies [17]. Moreover, inclusion of up to 20% of deoiled peanut meal flour resulted in acceptable product in terms of appearance, color, aroma, texture, flavor, and overall acceptability of bread [18]. The sensory results indicate that the panel members liked the peanut flavor and aroma [57].

### 3.6. Principal Component Analysis

**Figure 1:**
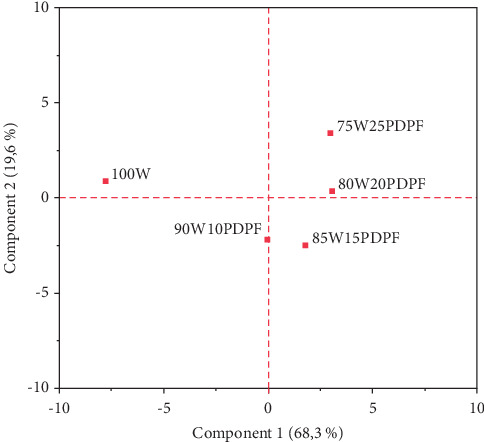
Score plots of samples with different proportions of wheat and PDPF (100 W = 100% wheat; 90W10PDPF = 90% wheat and 10%PDPF; 85W25PDPF = 85% wheat and 15% PDPF; 80W20PDPF = 80% wheat and 20% PDPF; 75W25PDPF = 75% wheat and 25% PDPF).

**Figure 2:**
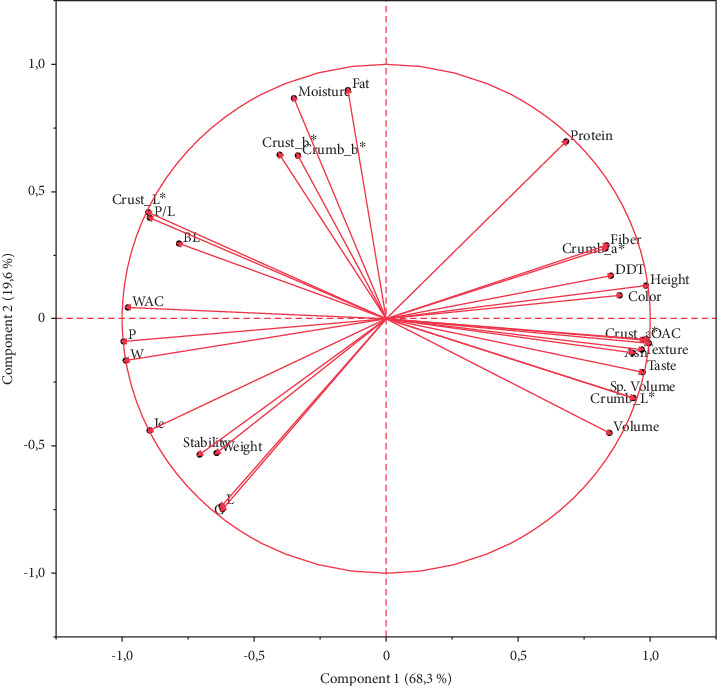
Loading plots showing the association between variables of muffins.

The association between variables was analysed using principal component analysis. The results indicated that the first three components explained about 96.1% of the variabilities where the first, second, and third components explained 68.3%, 19.6%, and 8.2%, respectively. The score plot (Figure 1) shows a visual presentation of the different samples exhibiting how the samples are related. The score plot indicates that the control (100 W) is negatively associated with the samples 75W25PDPF and 80W20PDPF in the first principal component indicating that functional properties of the flour and the physical properties and sensory attributes of muffins made from 75W25PDPF and 80W20PDPF have similar properties. On the other hand, the 90W10PDPF and 85W15PDPF are positively associated in the second principal component indicating that the samples have similar properties. The loading plot (Figure 2) shows the association between variables. The sensory attributes (texture, color, flavor, taste, and OAC) were positively associated with muffin physical properties (volume, specific volume, height, crumb_*L*, crumb_*a*^∗^, crust_*a*^∗^), proximate composition (protein, fiber, and ash), and farinograph property (DDT). All the alveograph properties were negatively associated with the sensory and physical properties of muffins (volume, specific volume, and height).

**Figure 3:**
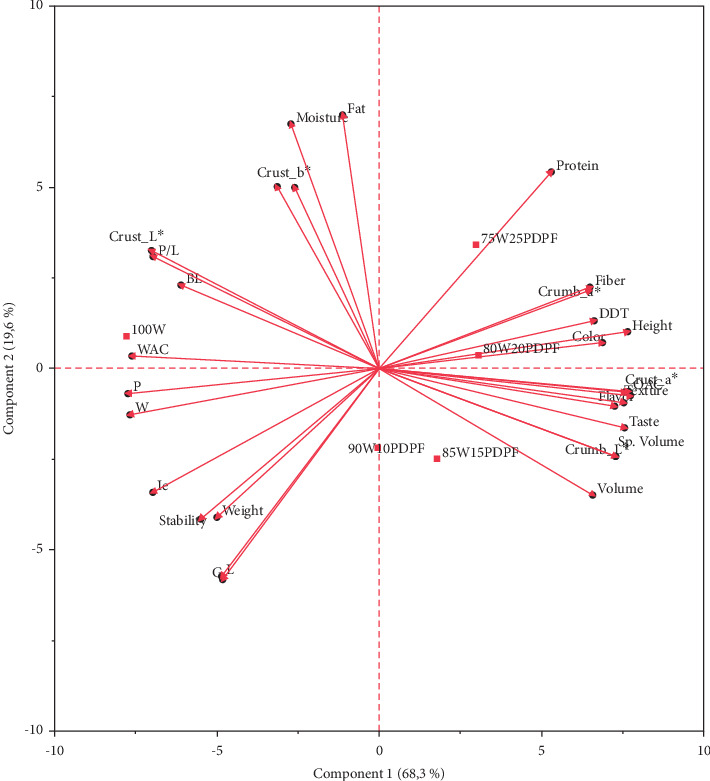
Biplots showing the association between samples and variables of muffins (100 W = 100% wheat; 90W10PDPF = 90% wheat and 10%PDPF; 85W25PDPF = 85% wheat and 15% PDPF; 80W20PDPF = 80% wheat and 20% PDPF; 75W25PDPF = 75% wheat and 25% PDPF).

The association between the composite flour formulation and the dough rheological properties, physicochemical properties, and sensory attributes of muffins is depicted by the biplot (Figure 3). Samples with high percentage PDPF (75W25PDPF and 80W20PDPF) are positively associated with the sensory attributes (including texture, color, flavor, taste, and OAC), physical properties (volume, specific volume, height, crumb_*L*^∗^, crust_*a*^∗^, and crumb_*a*^∗^), proximate composition (protein and fiber), and DDT. On the other hand, the control is positively associated with alveograph properties (*W*, *P*, *P*/*L*, and Ie) and farinograph properties (WAC and stability) indicating that wheat flour dough is more workable than a weakened composite flour dough [8, 32, 36]. The association between high PDPF, sensory attributes, crumb_*L*^∗^, crust_*a*^∗^, and crumb_*a*^∗^ is indicative of the attractive appearance and unique flavor imparted by the peanut flour [18, 57]. The association between high PDPF with ash, protein, and fiber is also an expected association since peanut is a good source of protein, fiber and minerals [14, 17, 18, 57].

## 4. Conclusion

The Farinogpah and Alveograph properties were significantly (*p* < 0.05) affected due to the proportion of PDPF. The DDT increased, whereas the WAC, stability, *P*, *W*, *L*, *G*, Ie and *P*/*L* significantly (*p* < 0.05) decreased with increase in the proportion of PDPF. Samples with high percentage PDPF (25% PDPF and 20% PDPF) were positively associated with the sensory attributes (color, texture, flavor, taste, and OAC), physical properties, proximate composition (protein and fiber), and DDT. Incorporation of the PDPF improved the sensory scores for taste, color, texture, flavor, and OAC. In view of the sensory scores, dough rheological properties, and the physical properties, PDPF can be incorporated up to 15% for acceptable muffins. Thus, the sample with the 15% PDPF is the best candidate in place of 100% wheat flour. The PDPF has substantial amount of fat which may compromise the shelf life. Further study may be conducted on the shelf life of the muffins and also compare it with muffins made from wheat and fully defatted peanut composite flour.

## Figures and Tables

**Table 1:** Rheological properties of dough from Farinograph and Alveograph tests.

**Treatment**	**DDT (min)**	**WAC (%)**	**Stability (min)**	**P** ** (mmH**_**2**_ **O)**	**L** ** (mm)**	**G** ** (−)**	**W** **(10** ^**−4**^ **J)**	**P**/**L (−)**	**I** **e** ** (%)**
Control (100%W)	2.10 ± 0.14^b^	59.65 ± 1.15^a^	11.40 ± 0.81^a^	107.5 ± 9.19^a^	60.0 ± 7.07^a^	17.20 ± 0.99^a^	241.5 ± 31.8^a^	1.80 ± 0.06^a^	54.15 ± 3.61^a^
10% PDPF	4.90 ± 0.31^a^	55.85 ± 0.78^b^	7.42 ± 3.06^abc^	65 ± 0.00^b^	63.5 ± 9.19^a^	17.70 ± 1.27^a^	136.5 ± 17.7^b^	1.04 ± 0.15^b^	43.35 ± 3.46^b^
15% PDPF	3.90 ± 1.56^ab^	55.90 ± 0.28^b^	10.80 ± 4.21^ab^	59 ± 1.41^bc^	56.0 ± 1.41^ab^	16.70 ± 0.21^ab^	108.5 ± 2.1^bc^	1.05 ± 0.00^b^	39 ± 3.82^bc^
20% PDPF	4.33 ± 0.15^a^	55.60 ± 0.71^b^	4.87 ± 0.28^bc^	47.5 ± 4.95^c^	43.0 ± 5.66^bc^	14.60 ± 0.99^bc^	72.0 ± 1.4^cd^	1.13 ± 0.26^b^	32.4 ± 3.39^cd^
25% PDPF	5.6 ± 0.85^a^	55.15 ± 0.49^b^	4.04 ± 0.48^c^	45 ± 7.07^c^	38.0 ± 2.83^c^	13.75 ± 0.49^c^	62.0 ± 8.5^d^	1.20 ± 0.28^b^	24.6 ± 3.68^d^

*Note:* Means in each column followed by different superscripts are significantly different (*p* < 0.05).

**Table 2:** Proximate composition of muffins made from wheat–PDPF composite flour.

**Treatment**	**Moisture content (%)**	**Protein (%)**	**Fat (%)**	**Ash (%)**	**Fiber (%)**
Control (100%W)	27.08 ± 0.22^a^	9.25 ± 0.06^d^	13.31 ± 5.43^b^	1.44 ± 0.17^c^	0.16 ± 0.05^c^
10% PDPF	24.38 ± 0.08^d^	9.99 ± 0.42^c^	17.85 ± 1.15^a^	1.74 ± 0.17^b^	0.34 ± 0.02^c^
15% PDPF	25.18 ± 0.09^c^	10.25 ± 0.22^c^	17.23 ± 0.47^ab^	1.92 ± 0.65^ab^	1.80 ± 0.02^b^
20% PDPF	25.95 ± 0.09^b^	11.15 ± 0.42^b^	17.67 ± 0.77^a^	2.04 ± 0.07^a^	2.11 ± 0.05^a^
25% PDPF	26.91 ± 0.07^a^	13.49 ± 0.54^a^	19.52 ± 1.70^a^	1.83 ± 0.17^ab^	2.04 ± 0.14^a^

*Note:* Means in each column followed by different superscripts are significantly different (*p* < 0.05).

**Table 3:** Physical properties of muffins made from wheat–PDPF composite flour.

**Treatment**	**Weight (g)**	**Height (cm)**	**Volume (cm** ^**3**^ **)**	**Sp. volume (cm** ^**3**^ **/g)**	**Baking loss (BL) (%)**
Control (100%W)	64.9 ± 4.25^a^	5.3 ± 0.48^a^	100.00 ± 20^b^	1.54 ± 0.29^b^	18.60 ± 1.98^a^
10% PDPF	63.4 ± 0.78^a^	5.6 ± 0.19^a^	128.0 ± 5.70^ab^	2.02 ± 0.06^ab^	15.20 ± 3.81^ab^
15% PDPF	65.0 ± 1.71^a^	5.6 ± 0.22^a^	145.3 ± 20.5^a^	2.23 ± 0.29^ab^	15.20 ± 1.27^ab^
20% PDPF	61.6 ± 1.88^a^	5.7 ± 0.11^a^	129.3 ± 17.01^ab^	2.12 ± 0.32^ab^	12.65 ± 0.21^b^
25% PDPF	62.0 ± 2.81^a^	5.7 ± 0.05^a^	127.0 ± 4.25^ab^	2.06 ± 0.20^a^	16.40 ± 0.42^ab^

*Note:* Means in each column followed by different superscripts are significantly different (*p* < 0.05).

**Table 4:** Crust and crumb color of muffins made from wheat–PDPF composite flour.

**Treatment**	**Crust color**			**Crumb color**		
	**L**	**a**	**b**	**L**	**a**	**b**
Control (100%W)	60.3 ± 4.8^a^	13.7 ± 1.8^b^	37.0 ± 0.34^a^	62.5 ± 0.0^ab^	4.2 ± 1.2^a^	25.2 ± 2.0^a^
10% PDPF	51.7 ± 4.9^b^	16.7 ± 2.6^a^	29.1 ± 5.70^b^	69.9 ± 7.6^a^	4.3 ± 1.6^a^	25.0 ± 1.7^a^
15% PDPF	51.1 ± 0.3^b^	18.9 ± 0.47^a^	33.6 ± 0.88^ab^	55.8 ± 2.1^bc^	5.3 ± 0.7^a^	22.3 ± 1.4^a^
20% PDPF	51.0 ± 5.2^b^	18.7 ± 0.63^a^	33.5 ± 4.02^ab^	51.2 ± 4.4^c^	5.5 ± 0.8^a^	23.5 ± 4.1^a^
25% PDPF	53.8 ± 0.3^ab^	18.4 ± 0.37^a^	35.3 ± 1.16^ab^	58.4 ± 0.5^bc^	5.5 ± 0.8^a^	25.7 ± 2.0^a^

*Note:* Means in each column followed by different superscripts are significantly different (*p* < 0.05).

**Table 5:** Sensory attributes of muffins made from wheat–PDPF composite flour.

**Treatment**	**Color**	**Taste**	**Flavor**	**Texture**	**OAC**
Control (100%W)	5.9 ± 1.58^a^	6.0 ± 1.74^a^	5.9 ± 1.76^a^	5.0 ± 2.38^a^	5.8 ± 1.63^a^
10% PDPF	6.8 ± 1.30^b^	7.0 ± 1.67^b^	6.7 ± 1.54^b^	6.5 ± 1.34^b^	6.9 ± 1.32^b^
15% PDPF	6.7 ± 1.42^b^	7.2 ± 1.44^b^	6.9 ± 1.35^b^	6.9 ± 1.32^b^	7.3 ± 0.97^b^
20% PDPF	6.6 ± 1.57^b^	7.1 ± 1.58^b^	7.0 ± 1.55^b^	6.6 ± 1.56^b^	7.2 ± 1.52^b^
25% PDPF	7.1 ± 1.32^b^	7.1 ± 1.60^b^	6.9 ± 1.69^b^	6.9 ± 1.60^b^	7.3 ± 1.41^b^

*Note:* Means in each column followed by different superscripts are significantly different (*p* < 0.05).

## Data Availability

The data that support the findings of this study are available on request from the corresponding author. The data are not publicly available due to privacy or ethical restrictions.
